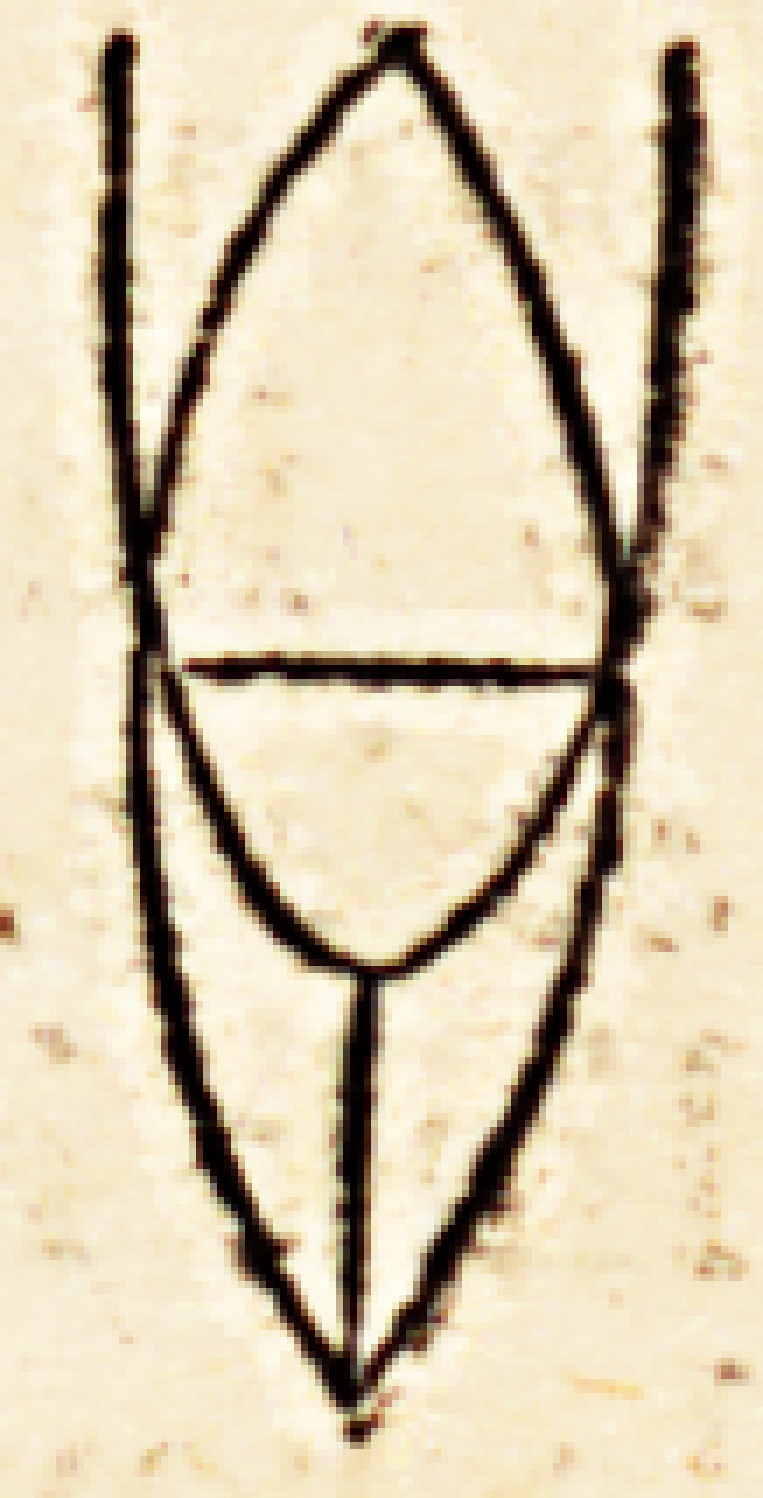# Mental Dynamics, in Relation to the Science of Medicine

**Published:** 1853-04-01

**Authors:** Stanhope Templeman Speer

**Affiliations:** PROFESSOR OF PHYSIOLOGY IN THE UNIVERSITY OF MONTPELLIER; CHELTENHAM


### MENTAL DYNAMICS, IN RELATION TO THE SCIENCE OF MEDICINE.

A COURSE OF LECTURES DELIVERED BY M. LORDAT, PROFESSOR OF PHYSIOLOGY IN THE UNIVERSITY OF MONTPELLIER. ARRANGED AND TRANSLATED BY STANHOPE TEMPLEMAN SPEER, M.D., CHELTENHAM.

### Lecture III.

Gentlemen,—It was stated in the last lecture, that from the moment of birth the human principle of intelligence undergoes a gradual expansive development, as does likewise the vital force. This simultaneous growth on the part of the two principles has been noticed by Lucretius; but the poet, wishing to prove that they were derived from the same source, and were of an identical nature, has allowed us to take for granted that they in reality ran a parallel course; such, however, is far from being the case, and I shall therefore endeavour to represent by certain lines the relative progress of zoonomic and intellectual existence, so as, if possible, to determine in what the essential causes of the two elements of life differ, and in what they agree. The respective lines employed for this purpose, while starting from one and the same point, will diverge, but they will not remain parallel. That which represents the vital force having, as will be seen, a certain start, it is necessary that the intellectual principle should undergo an acceleration through the intervention of favourable circumstances, such as education, study, &c. &c., in order that its representative line may regain lost ground, overtake and even outstrip its competitor. Thus, according to primitive disposition, to the mode of life, or to education, do we obtain an athlete, a hero, an academician, or a shepherd. True, it may happen that at a very early period the diverging lines which represent the principle of intelligence may outstrip those of the vital force; or that, on the other hand, they may remain closely united within the area of the diangle, up to the critical point—mind being thus buried in matter. Such variations, however, only serve to prove the non-identity of the two principles.

But what shall we say of the progress of the intellectual principle during those alternations of health, disease, and convalescence peculiar to zoonomic life? Should the former be represented by scolloped lines, such as we have shown to indicate the progress of the vital force? As a rule, no—facts do not allow of it; upon the supervention of the disease the two principles cease to run parallel, they no longer follow the same track, and consequently can no longer be represented by the same figure. In the scolloped diangle (mentioned in the last lecture), and intended to stand as an emblem of man's zoonomic existence, I showed you the vicissitudes to which the vital principle is subject; its alternations of strength and of weakness, its relapses, its dangers, its diseases and its recoveries; but when we attempt to unite the line which represents the life of the intellect with that of zoonomic life, we find that such unison is impossible. Allowing that disease may impede the natural development and the spontaneous budding (so to speak) of the intelligence, this latter principle can never be reduced by it to such an extremity as that to which the same disease has brought the vital force.

In instituting such a comparison, we recognise at once that this latter principle has in its proper essence suffered a diminution in quantity, of which the intellect is incapable. The principle of intelligence, conscious of its expansive powers and of its diverging action, cannot consent to any limitation, to any restriction, incompatible with its own peculiar nature.

I have here stated, however, what is the general rule. I have no wish to ignore the exceptions, should there be any. And we must allow that there are certain diseases in which the two powers, apparently co-operating with each other, would seem to be alike doomed to sterility, alike thrust out of their natural course; I allude now to what is denominated mental alienation. Do such cases authorize us to assert that the life of the intelligence, like that of the animal, is liable to alternations of debility, convalescence, vigour, &c.; and that the figurative line by which it may be represented, should, as a consequence, be festooned thus  ? I should hesitate before coming to such a conclusion. The theory of mental derangements is up to the present moment more or less obscure. Before the eighteenth century the history of these diseases was too incomplete to hope for any satisfactory physiological deductions capable of being derived from them. Since then, however, facts have become clearer and more circumstantial, but, unfortunately, Organicism has contrived to hamper this part of our science. It is indeed much to be wished that physicians should be undeceived, and diverted from what are too often merely anatomical delusions, convinced of the duality of the human dynamism, and capable of demonstrating what is the part played by the intellectual principle in these sad scenes. Is it really the subject of an affection comparable to those which disturb the vital force, compromise its existence, render it incapable of properly executing its peculiar functions, and then subject it to a process of convalescence which, after all, is but an imitation of adolescence? Or, on the other hand, is the principle of intelligence in a similar condition to a troubled sleep, in which, by the establishment of a truce between the two agencies, the vital force ruptures those legitimate bonds which should naturally exist between the intellectual principle and the external world, leaving the latter devoid of all guidance, liberty, or logical capability.

If this were really the case, the intellectual principle would be neither active nor passive, nor would it be liable to erratic deviation or dangerous prostration.—After such a species of hallucination, its figurative line could no longer follow the inflexions of the curve alluded to in the last lecture.

Now this appears to me to be exemplified in the disease with which our unfortunate king Charles VI. was afflicted. You are aware that it consisted in a furious but intermittent mania, the paroxysms of which were often of long duration—such as six or ten months, while the intervals between them were short. After the first attack, which had lasted six months, "the king recovered—awoke as it were from a dream," says Anquetil, and was much astonished and grieved at what had taken place during what might be termed his "absence;" proving that the intellectual past was connected with the present, just as when in health the past of yesterday is linked with the present of to-day, in spite of the intervention of sleep. After the attack which he suffered in 1403, when 35 years of age, Charles VI., says the same writer, "took advantage of a lucid interval to fix upon a settled form of government. Up to this period his arrangements had merely been provisional; but the king now gave to these all that solemnity and importance which might render them permanent." You perceive, therefore, that when reason returned the intellect did not require re-educating. It appeared as vigorous and sound as at the moment when the attack supervened; nay, it had even improved, since its own calamity had proved its instructor. As long then as I entertain a doubt upon these momentous and practical questions, so long shall I avoid compromising the truth, and must consequently refrain from representing such aberrations by lines similar to what I employ in figuring the irregularities to which the vital force is subject. I prefer breaking off such lines at the points which correspond to mental alienation, taking them up again at those which correspond with the return of reason.

Let us now revert to what Galen has termed accelerated or anticipated senescence. You are perhaps aware that he designated as old age or senescence, all tendency to death, irrespective of age, "*Senium est vita ad interitum.*" We have already represented this by the annexed figure.

In it we perceive a premature inflexion of the lines which represent the vital force, such inflexion rendering the tendency to death, or, in other words, the progressive declension of the vital force, more hasty or more rapid; but can we, in representing the life of the intelligence, employ similar figures indicative also of its premature decay? Certainly not; materialists themselves dare not deny the insenescence of the intellectual principle in cases of premature old age. They mention it not, and even allow that under such circumstances the dying man often gives evidence of reason, talent, or genius, scarcely manifested before, and what has been proverbially denominated the *swan's song*. They have not, however, learnt that this divergence of the lines which represent the life of the intellect contrasting with the convergence of those which represent the animal existence, thus—upsets at a blow the Epicurean theory.

The number of men who, like Pergolesi, Gilbert, Mondonville, Mozart, Drouais, have died of a languishing disease, in the prime of their intellectual faculties, is too considerable even to make choice of a few, as examples. But we have so recently to deplore a loss of this description, that I cannot allow it to pass over in silence. M. Casimir Delavigne has just departed, at the close of a premature old age, and while engaged in a work requiring the greatest possible amount of intellectual perfection and integrity. A friend who witnessed his end, has given an account of it in the following words:—"C. Delavigne, at the time of his demise, was on his road to Montpellier, his physicians having held out to him a hope of cure in a less rigorous climate. On his arrival at Lyons, on the day of his death, accompanied by his wife and son, he became somewhat discouraged by the fatigue he had undergone. A physician of the town assured him, however, of restoration to health, and encouraged him greatly, while he at the same time informed his wife that he had but a few hours to live. Such was, nevertheless, the illusion under which the invalid laboured, that he wished to start again immediately, and merely consented to retire to bed from a wish to please his sorrowing relatives.

The fatal moment took place at 9, p.m. "At 8 o'clock, in order to divert his son, M. Delavigne requested his wife to read aloud from one of Sir Walter Scott's novels; she did so for three quarters of an hour. At a quarter to nine, he asked for something to quench his thirst, and as his wife, in order not to fatigue him, took some little precaution in offering the glass, the poet exclaimed: "*Give it me; give it me; I am strong!*" and raised himself into a sitting posture upon his bed; then, leaning his head on his hand, he requested Madame Delavigne to continue reading. But death was at hand; the countenance of the sick man was already undergoing a change. His wife perceived it, but repressing her grief, she read in a voice rendered unintelligible by emotion: "How!" exclaimed the poet, "*you are omitting entire phrases;*" then addressing his son, he ordered him to read instead. In another minute the head of Casimir Delavigne fell back on the pillow; he then began to recite aloud some lines from a tragedy at which he had been for some time engaged, and which was to have been styled "Melusine," and ten minutes after, the author of "Les Messeniennes" was no more; the work which occupied his last thoughts perishing along with him, for it is well known, that Casimir Delavigne never wrote his pieces until they were completely finished, reciting them by memory at the moment they were to be handed over to the theatre."

It is therefore sufficiently evident, that in this premature, or, so to speak, Galenic senescence of C. Delavigne, the vital force was on the eve of destruction while the intellectual powers were in full operation. He was instructing his son, complaining of a want of succession in the ideas to which he was listening, finding fault with his corporeal organs and with that vital force now no longer obedient to his dictates, and then consoling himself by having recourse to poetry, or, in other words, to one of the most exalted operations of which the human mind is capable, up to the last moments of his existence. Judge then from this, if in such cases it be proper to approximate those curved lines which we have made to represent the intellectual life of man.

We now arrive at a point to which I have been long looking forward; we are at the apogee of the vital force, and, as a consequence, upon the threshold of normal old age. What now becomes of the intellectual principle? I have already answered the question;—it does *not* undergo progressive declension. In other words, I abstain from giving to the lines which represent mental existence such an inflexion as would lead to their junction.

Such is the assertion which I must now endeavour to prove, in opposition to those which the materialists maintain, and of which the following are specimens:—"At the close of the period of maturity," says Cabanis, "there occurs a gradual decomposition in the humours of the body, to which succeed gout, stone, rheumatism, apoplectic predisposition, &c. Sometimes the acrimony of the humours excites a species of nervous reaction upon itself, and produces momentarily a renewal of youth, but *soon the old man becomes evident, acting and thinking with difficulty*, caring only for himself, and seeking but for that repose which is destined to close this melancholy condition. . . . . Again, we may remark, that in old age the weakness of the brain and of those operations which emanate from it, impart to their objects the same uncertainty and the same characteristics which they presented during childhood. The extremes resemble each other."

To render the above propositions, so necessary to the support of materialism, at all tenable, we must show that the decay of the intellectual faculties, and the renewal of childhood's characteristics, are not accidental events, resulting from a variety of diseases such as have been noticed at all periods of life . . . but a constant and unvarying termination of old age, and that senile death is always preceded by a state of imbecility, as it certainly is by a decline of the vital force, and by progressive incapacity on the part of every corporeal function. Without such a condition, the materialist theory is absurd. Facts, then, are distinctly opposed to such assertions, for—1st, It is not true that the intellect becomes infallibly weaker after the vital force has passed its culminating point; 2nd, it usually happens, that the understanding acquires more strength during the first half of that period which we designate as old age; 3rd, it is impossible to assign any period of existence, at which the reasoning powers naturally suffer deterioration; and 4th, nothing is more common than to behold senile death; that is to say, the natural extinction of the vital force; having as an intelligent spectator and witness, the intellectual principle, its congenitor.

Let us here remark, that the opinions of Lucretius and his followers, relative to the decay of this principle during the decline of life, are not derived from the fathers of medicine.

Hippocrates has taken cognizance of the infirmities and diseases of the aged, and enters into a detailed account of the imperfections which supervene, relative to their sensorial capabilities, without, however, saying anything of intellectual decay. Not, indeed, that he believed them to be exempt from imbecility, eccentricity, or mental alienation, to all of which the human race are liable; but he rather looked upon such diseases as eventualities or accidents, and not as the natural and infallible termination of the human understanding.

Without doubt, we may consider Galen as one of those who have laid the most stress upon old age and its inconveniences. In his numerous works on physiology, hygiène, pathology, &c., he never loses sight of it for a moment, but notices all the successive deprivations which the vital principle suffers, the alterations in the chemical constitution of the fluids and solids, the breaches made in various organs, their diminished functional activity, the morbid sensations resulting therefrom, the enfeebled movements, the sense of debility, the absence, or what is worse, the vitiation of the appetites and of the instincts, the perversion of the vital affections and tastes . . . . . nothing is wanting to the picture, if you only take the trouble to study its different items in the Medical Encyclopedia of its laborious author. But observe, that in this description of old age, Galen (who, although a Deist, had a decided leaning to materialism, and to the mutability of the intellect) does not attempt to add to the foregoing characteristics an enfeebled condition of the latter principle. He indeed says, that the aged "have but little capability to execute any voluntary act;" but none deny this; the proverb which we daily hear, "*Could youth do what age knew*," is but an expression of the fact. They have the will, they have therefore intelligence. If the will be not obeyed, it is not the mental powers that are to be found fault with, but the vital principle, now too debilitated to answer the solicitation, or to obey the injunction.

Galen has not failed to observe in the aged a very characteristic trait—namely, a weakening of the memory, of so common occurrence as to be almost considered infallible. But this does not prove a weakening of the intellectual principle. I have on another occasion, in my lectures on the transmission of thought, already shown that the memory (or the preservation of ideas in their full integrity) and the *rememoration* or *re*-collection of these ideas, with their manifestation to the powers of thought, are complex functions, executed in concert by the two principles. The remembrance of a fact is usually composed of two elements, the one concrete, the other abstract. The first of these is rather the offspring of the vital force than of the intellectual principle. It is not surprising, therefore, that the aged condition of the former should manifest itself, while the latter power preserves its full integrity. Rememoration being thus an operation in which the whole human dynamism is concerned, a certain degree of oblivion must always be noticed in the memory, since age has of necessity produced its effect upon one of the co-associates.

I would therefore request you to bear in mind those characteristics by which we estimate the value of the intellect. It is impossible to attribute to it a process of decay until there be a weakening of the judgment, of reason, of the appreciation of ordinary events, of the power of abstraction, or of the faculty of logically combining ideas, which, whether abstract or concrete, are the offsprings of the natural psychical tendencies of the individual. As long as these faculties preserve their ordinary condition there can be no mental senescence.

Among the problems of Aristotle we find the following:—"Why is it that in youth we learn more quickly, and wherefore is it that as age advances the intellect becomes more powerful? *Cur seniores amplius mente valeamus, juniores citius discimus?*" It is unnecessary now to demand a reason for such assertions; but it is evident that the philosopher looked upon the facts they express as above all doubt, and this it is which would lead to a belief that the dogma relative to the senescence and normal decline of the intellectual principle must have arisen and have been fostered solely by the sect of the materialists.

But in order that the principle of intelligence should participate in the senescence of the vital force, it must be shown that after this latter has attained its apogee, the intellectual faculties at once deteriorate, while the productions of the mind, subsequent to this epoch, become daily less profound, less perfect, or, in other words, that they retrograde. It would require that the "Femmes Savantes," composed ten years after the culminating point (40 years) of the author's vital principle, should bear evidence of inferiority of intellect to the "Etourdi," composed seven years before its culmination: that "Athalie" should be inferior to "Alexandre," that the "Spirit of the Laws" should evince less power of understanding than the "Lettres Persannes." It would require that the works of Kant composed at 40 years of age should much surpass those which he published at 60. He himself, in reality, blushed at the remembrance of the former, while the public have adopted only the latter. If the later productions, then, possess more value than the earlier ones, it becomes impossible to say that the authors have aged *intellectually* since the commencing declension of their vital force.

Let us then avoid, by all means, the doctrine of the senescence of the intellectual principle, since the understanding is never so fertile, never so vigorous, as after the culmination of the vital force.

M. Chateaubriand, doubtless, did not believe in a senescence of the intelligence, isochronous with that of the vital force; since, in speaking of the death of Madame de Stael, which occurred at the critical age of this illustrious woman, he says, "We cannot too much regret the premature end of Madame de Stael; her talents were on the increase, her style became purer in proportion as youth weighed less upon her, while her ideas were gradually disengaging themselves from their corporeal tenement, and partaking more and more of immortality. (1)" (*Etudes Historiques*, Preface.)

The celebrated Father Sirmond, whom Naudé designated as "*an inexhaustible treasury of ecclesiastical lore,*" likewise entertained no doubt but that the intellectual principle acquired an increase of power when the vital force was already on the decline. He advised all sages to postpone committing their thoughts to paper until the age of 50, and he himself adopted the precept, having attained to 52 before he determined to become an author. And then, as if to prove that years could not daunt his intellectual powers, he continued to write for 40 years. The vital force was extinguished within him (but not by disease) at the age of 93, and his understanding, which had never failed, then only yielded to the inevitable decree.—(*Life of Father Sirmond by Colomiez.*)

This integrity of the intellectual principle is not limited to certain points of the human understanding. Authors and artists of every description have often presented examples of it. Our own painter, Vien, continued to exercise his art up to the period of his death—that is, up to 90 years of age. Solimène did the same up to 84, at which epoch an accident rendered him sick and infirm. His last production was worthy of all praise. He was, moreover, a poet. "It was a matter of wonder," says his historian, "that at the age of 80 his memory enabled him to select the choicest specimens from the different poets, and to apply them in the happiest manner." At 88 he became both blind and deaf; "and during this period of his existence he was visited by his pupils, who, by his expositions relative to the difficulties of their common art and the means of surmounting them, profited as much as by seeing him actually engaged in its practice. He was in the habit of telling them, that being deprived of all corporeal vision, he could see more clearly with the mind's eye than when able to represent on canvas."—(*Abridgment of the Life of Celebrated Painters, etc., by M. d'Argenville.*)

You may possibly remember what M. Raoul Rochette has lately said relative to the last days of the composer Cherubini, whose conversation at the age of 82 was as brilliant as during the meridian of his existence.

Gossec, at the age of 78, composed a *Te Deum,* considered by connoisseurs as one of his best productions. "Galuppi, the master of Sacchini, retained up to an advanced age (82) all the vivacity and gaiety of his youthful days, both in his disposition and in his works. It is even said that the taste, talent, and imagination, of which he gave evidence in his last operas and church compositions, caused them to surpass all those which he had published during the preceding period of his existence."

The Jesuit Sirmond deduced from his own observations, and proved by his works, that the most solid and durable productions of the intellect emanated from their authors at a period of existence when the vital force had passed at least the first quarter of its progressive declension—*i. e.*, between 40 and 50 years of age. The great Corneille certainly could not have imagined his intellectual powers to be of less calibre at 70 than at 30 years of age. "Although covered with laurels," says the Abbé Raynal, "Corneille would not allow that the hour had arrived for him to withdraw from the eyes of the world; and in a poetic address to the king, he assures him that while fashion may have diverted the popular taste into another channel, if he, the monarch, would but reiterate those professions of regard which had formerly so inspired his poetic muse, he himself would be fully capable of recommencing and of composing lines equal in every respect to his former ones."

II. The facts just narrated, suffice, I think, to show that the intellectual principle does not necessarily undergo a process of decay after the culminating period of the vital force. This assertion requires to be reinforced by proofs, which I purpose to adduce, of the truth of the following proposition:—

*That it is not possible to assign a particular period during old age at which the understanding is of necessity prone to decline.*

Nothing, indeed, is more common than to witness aged individuals preserving all their intellectual faculties up to the close of a most protracted existence, although time may have made all the corporeal ravages which it is accustomed to inflict. This resistance of the mind to the senescence of the vital system becomes then a problem in the eyes of the physician. It would appear to *us* a prodigy, did we not always bear in mind that "*solidarity*" which exists, not only between various organs, but likewise between the two great principles of the human dynamism—a solidarity of which I have often reminded you, and which constitutes one of the great fundamental principles of Medical Physiology.

Instances of the insenescence of the intellectual principle are so numerous, that I have but a difficulty in selecting them. If, therefore, I confine myself to examples furnished by historical characters, it is but for the dignity of public instruction. There can scarcely be an individual, who is not capable of citing, whether in his own family, in the place of his birth, or even in the street he inhabits, several examples of this kind. I do not wish, however, to adduce those hoary sages of remote antiquity, who ceased not to think and write till the hand of death was upon them; such as Plato, Chrysippus, Carneades, Varro, Isocrates, Sophocles. I might by so doing expose myself to discussions relative to the exact duration of their lives. We find a sufficient number of examples in modern time of similar individuals, whose first and last moments have been accurately and legally chronicled.

I might here allude to several centenarians, of whom the journals have ever and anon made mention, and who were full of life and intelligence when we last heard of them; for instance, M. des Quersonnières, 116 years of age, now residing in Paris, an accomplished poet, remarkable for his powers of conversation, and full of vivacity. Again, I might adduce M. Leroy, of Rambouillet, who at the advanced age of 100, composed a remarkably beautiful and spirited poetic effusion. But I shall probably be told that such insenescence is merely provisional, inasmuch as they still live and enjoy good health, and that we should await their decease before asserting that their intellectual powers have resisted the various stages of decay; I must therefore confine myself to those cases in which the understanding has remained unscathed up to the period of vital extinction. Be it so: I will restrict myself to such.

The life of Fontanelle is one upon which I am prone to lay much stress; inasmuch as his character has been, as it were, constantly before the world; while all the details connected with his life are authentic. In him we may follow, step by step, the phases of increase and decrease which the vital force underwent, and at the same time observe the insenescence and immutability of the intellectual principle. Should we fail to perceive any sensible augmentation of its brilliancy, it would be at least impossible to discern any dimness in that very temple of light. You are aware that he died a centenary. Hear what has been said of him a short time before and after his decease:—"The intellectual faculties," says the Abbé Trublet, "with the exception of a slight defect of memory, had preserved their integrity in spite of corporeal debility. His thoughts were elevated, his expressions finished, his answers quick and to the point, his reasoning powers accurate and profound."

The duality of the human dynamism, the decay of the one principle and the permanence of the other, are especially remarkable in a correspondence between Fontanelle and the Cardinal de Fleury his contemporary, who governed France as prime minister from the age of 70 to that of 90. When near 80 years old, Fontanelle, feeling the need of physical repose, wrote to his eminence, asking permission to vacate his post as perpetual secretary of the Academy of Sciences. The prime minister, knowing something of the effects of age, since he himself had attained that of 84, refused his request, but in the mildest terms. Three years afterwards, Fontanelle reiterated his demand. This time the Cardinal answered in his own handwriting, "You are but an indolent, lazy fellow," says he, "but we must, I suppose, occasionally indulge such characters." In truth, on the one hand, the seventeen years of idleness and liberty in which the suppliant indulged, justified the accusation brought against him by the prime minister; while on the other, his deafness, impaired vision, general debility, gouty disposition, &c., sufficiently exonerated him for indulging in repose and independence.

All who now hear me are aware of what Voltaire was, when, at the age of 84, he came to Paris, to "*seek a triumph and to find a tomb*," as he himself said. The account of that journey sufficiently shows that he was still, what he had been during the last forty years.

Marshal Richelieu, who died at the age of 93, could only be considered aged in relation to the vital force. Up to his latest breath, his mind, so pre-eminently the reflection of the epoch, remained perfect. His last words, uttered but a few minutes previous to his departure, were characteristic of that gallantry of which he had all along been a model. His daughter-in-law, wishing to encourage him, said, "You are not so ill as you would wish us to believe; your countenance is charming." "What!" said he, with the utmost vivacity, "*has my face been converted into a mirror?*" Was it possible to say with more delicacy, that the charming countenance she beheld was but the reflexion of her own?

Another example presents itself to my mind, which I cannot omit to mention. The Count Simeon, who died at the commencement of the year 1842, was born in 1749. His panegyrist, M. Portalis, when delivering his funeral oration in the Chamber of Peers, says of him: "About fourteen months have now elapsed since he expired without suffering. His death came upon all but himself, as an unexpected blow, so much had they become habituated to seeing him live without scarcely appearing to grow old." During the above discourse, M. Portalis, in alluding chronologically to the events of the Count's life, says of the year 1838, "This year was to him pregnant with remembrance of the past. In the spot I now occupy, he pronounced the eulogy of an illustrious peer, a nonogenarian like himself; and who, after sharing his labours and proscription, had preceded him to the tomb. If Homer's description touches us, where he describes the hoary Nestor bidding farewell to the youthful heroes destined to survive him, is there not something especially solemn in the spectacle of this aged man paying the last tribute of affection to the worthy companion of his own career and its concomitant perils, and uttering with a calm, sad voice over his tomb, those sublime words so soon to re-echo over his own? At all times solicitous for the dignity of this chamber, he in the year 1840 suggested a proposition for the amelioration of its internal arrangements."

In our own epoch there is perhaps no man who has furnished so many proofs of the insenescence of the intellectual principle during corporeal old age, as M. Lantier, author of the "*Travels of Antenor*," "*Travels in Spain*," "*Travels in Switzerland*," besides many poetical works; he died at Marseilles in the year 1826, aged 92 years. At 91 he offered a parting tribute to the muses, in publishing "Geoffrey Rudel, or the Troubadour;" a poem in eight cantos.

His historian says of him, "He has written and repeatedly asserts, 'that they are but fools that grow old.'" In saying this, however, it must be understood that he alludes merely to the principle of intelligence; since as regards the senescence of the vital force, he sufficiently recognises and acknowledges it in a letter written eighteen months before his death, and of which a *fac simile* has been published. His trembling hands, his failing vision, his wandering but continual bodily pains, his complaints against a condition which permitted him neither to live nor die, and his playful determination and vow, at once to quit this earth, are all the expressions of conscious decrepitude. In reading this epistle, it is impossible not to be as much struck with the endurance of the one principle of the dynamism as with the decay of the other.*

As regards the exception made by M. Lantier relative to those whom he designates "fools," we must enter our protest against it. It appears to be as arbitrary a decision as that of Spinosa against philosophers on the one hand, and the vulgar-minded on the other. To the former he granted immortality, to the latter annihilation both of body and soul. I may be permitted to entertain doubts relative to such off-hand expressions, inasmuch as the truly intellectual are unfortunately somewhat prone to designate as fools all who do not think along with them.

For my part, I have not perceived this change for the worse after the lapse of time, in those whose intellectual powers were below the average. Their mind was the same, and they had merely lost those corporeal attractions which had served to veil its imperfections in youth. I hear, for instance, that such and such a female, 60 years of age, dotes. On visiting her, I find her conversation the same as it was 40 years ago, when she was considered beautiful, vivacious, worthy of all admiration. In youth, she had borrowed with compound interest from her lovely countenance wherewith to counteract the deficiences of the mind, and now that the pledge has lost so much of its value,

* It were impossible at the present moment, and while engaged in considering the insenescence of the intellectual principle, to pass over a most prominent example, occurring in one whose fame had rendered him the cynosure of his native land, and whose every action was looked upon with interest by a grateful country. Within the last month England's greatest military hero has paid the debt of nature at the advanced age of 83. In the person of the illustrious dead, the great Duke of Wellington, we have indeed strong evidence of the duality of the human dynamism, and of the relative value of its component parts during the latter years of man's existence. In him the decay of the vital principle, spite of his general good health, his abstemious habits, his Spartan-like simplicity of life, and his rigid attention to the welfare of the body, had become during the last few years but too evident. Whether on foot or on horseback, at the review, the chamber of state, or the brilliant assembly, it was plain to all who beheld him, that the conqueror of Europe's scourge—he who had stood on the eventful field of Waterloo in the full bloom of manly vigour and perfection—the restorer of a dynasty, the herald of a long-continued peace to the trampled-down, blood-stained nations of Europe—was now an aged, infirm, grey-headed man, whose trembling hands could scarce support that sword of state, which the veriest stripling might have wielded with comparative ease. But if such were the condition of the vital force during the closing scenes of the great duke's life, what shall we say of the intellectual principle? Did this afford similar proof of rapidly-approaching annihilation? Did its aged possessor in aught realize the assertions of Cabanis? Did he aspire only to a termination of his career? Did he think or act with difficulty? Was he regardless of all but of himself? The records of the last ten years—nay, his speeches and actions during the past twelve months—abundantly refute such suppositions. That his mental activity was still a prominent feature in his character is evinced by the interest which he took in objects of so dissimilar a character, as a university commission on the one hand, and in the regulations for the militia and transport of soldiers by railway on the other—matters which would appear to have engrossed his attention up to the very portals of the tomb. That his powers of judgment and foresight were unimpaired, is evident from his last speech on the Militia Bill. His Grace, it is said, spoke with difficulty, and the long pauses between his sentences, the creditors have thought better of it, and will no longer believe in the solvent condition of that intellect upon which they had calculated, and which nevertheless has lost but the value of the security.

III. Too much is said respecting *senile imbecility,* to neglect a further examination of the subject.

If we carefully study the facts which have been made to serve as texts for this fiction, we shall find that they are in reality, cases of accidental idiotism, to which we all are liable at any period of our existence. A progressive idiotism of such a nature as to bear out this doctrine has never been witnessed. Were an observer worthy of confidence to tell me of a man who had lapsed into this vegetative condition, in as gradual and continuous a manner as occurs in the case of the vital force, I would accept the fact, but I could not admit of its explanation, as being founded upon a normal progressive extinction of the intellectual principle. I should consider it rather as an accidental disease, inasmuch as the senescence of this said principle ought to be as common as constant, and as infallible as that of the vital force.

I know not from whence Richerand has drawn his ideas relative to the soperose condition of the aged. There is a poetical proverb which asserts the contrary, and tells us that the tendency to sleep is as much a morbid symptom in their case as is wakefulness in youth. Richerand would have us believe that this condition is the result of an obliteration of the ideas. The morbid sleep of the famous Moivre does not, however, support such a theory. "Towards the close of his career, he lost his hearing and his eye-sight, and the necessity for sleep increased to such a degree that he required at least twenty hours of it." It is not, however, said that during his four waking hours he had lost ought of his mathematical genius, or of that excellent memory which allowed him to repeat "entire scenes from the 'Misanthrope,' with all the emphasis and intonation with which he remembered to have heard them delivered sixty years before, at Paris, by Molière's own company."

and sometimes between the very words, betrayed the effort it required to proceed. But while thus struggling against the results of vital incapacity, his remarks, it is added, were, as ever, fully to the point; and none would appear to have taken so rational, so liberal, or so unimpassioned a view of the militia question, in its ultimate bearings upon national defence, as the veteran but decrepit soldier, who once more raised his voice in behalf of his country's welfare. On this, as on all previous occasions, his opinions were received with a respect and consideration which in itself constituted a tacit acknowledgment, a silent homage to the intellectual principle manfully struggling amid the crumbling ruins of its earthly tenement. Such homage, however, was not confined to the august assembly upon whose ears his final accents fell; it was universal; it was even innate in the breasts of Englishmen. Has a foreign invasion been uppermost in their minds, the fact that the great duke was still alive to direct his ancient paladins, and head his country's small but gallant host, too often sufficed to quell a salutary fear; and could we suppose such a calamity to have been really imminent, to whom would England's sons have looked with such anxiety, with such hopeful anticipations of success, or such visions of domestic security, as to the venerable, grey-headed warrior, whom all acknowledged to be, as of old, the trusty guardian of his country's honour, the chiefest warrant for a continuance of her inviolate condition. But, again, when the demon spirit of intestine insubordination had given evidence of a mighty but slumbering eruption, to whom did the partizans of loyalty and order look, but to the aged hero, who with all the alacrity and freshness of youth had at once taken upon himself the protectorship of the world's metropolis, and whose arrangements upon that memorable occasion were, for efficiency and completeness, worthy of the same master mind that had once planned the lines of Torres Vedras. It was enough, on the 10th of April, 1848, to know that the duke had taken the matter in hand, and was again at the head of his troops, for each citizen to feel that all was done, and would be done, that could be done; and in this additional acknowledgment of the veteran warrior's intellectual and moral superiority, we may again recognise an indirect tribute to that mental insenescence of which our author was so ardent a supporter, and of which he himself, while delivering the above lectures at the advanced age of 82, afforded so striking an example. S. T. S.

In truth, can we confound accidental disease with the natural tendencies of the intellectual principle? No; setting aside mental affections, the theory of which has no relation to decay, the aged, as aged, are not imbecile. A man may become as blind and deaf as Moivre; his hand may tremble to such a degree as to prevent him tracing a line or even a character; his organs of speech may refuse to give utterance to a single word; his memory may fail to recal the sounds which were wont to express his thoughts, as may be noticed in healthy-minded persons suffering from verbal amnesia; but with all this, he will not resemble the idiot, since he may still possess the whole system of ideas resulting from and acquired by a long existence, and may combine them intentionally, as before he had lost the power of verbal expression.

In opposition to misconstrued facts, I shall adduce the following:

In the solemn audience of the Court of Cassation of the present year, M. Dupin, procurator-general, pronounced the eulogy of M. Etienne Pasquier, author of "Researches on France." This celebrated lawyer of the sixteenth century, after having for a long period discharged the duties of king's counsel, retired from his post at the age of 74, in order to give up his time and energies to the pursuit of literature; and to these his favourite studies he devoted the remainder of his existence up to the period of his death, which took place on the 31st of August, 1615, at the age of 86. It has been remarked as a singular fact, *that he closed his own eyes previous to the fatal moment.* Again, history has handed down to us an anecdote relative to the death of Julius Cæsar, where the mighty conqueror, falling under the blows of his murderers, calmly arranges his toga, in order that his fall might be decent. The fact is worthy of remembrance in a moral point of view; such presence of mind, and such resolution under such circumstances, are of rare occurrence even in a man 56 years of age. But the previous observation, made in connexion with the natural extinction of an individual of 86, is truly a precious anthropic phenomenon; do we not see in it a hidden principle, preserving all its consciousness, witnessing the destruction of the vital system it had hitherto inhabited, and even performing the funeral rites of its own host?

Facts analogous to the above are to be found everywhere, and my sole diffi culty is to keep within proper limits. I cannot, however, omit to mention the following example, on account of the remarkable expression of the dying man himself. The individual in question is Pierre Louis Anquetil, no less remarkable for his own intrinsic merits, than for the additional proof which he affords of the truth of my argument. I shall quote a short passage from his biography:—"Anquetil had attained his 84th year; his robust constitution and moderate habits having preserved him from the ordinary infirmities attendant upon old age. While still meditating great achievements, he was attacked by erysipelas of such severity, that his physician at once perceived his end to be nigh at hand, and informed him thereof. Anquetil heard his doom with the resignation and tranquillity of a philosopher, and said to those who came to take their last farewell, "*My friends, you behold a man dying, full of life.*"

The prognosis of his medical attendant was but too soon realized; the disease gained ground; and on the 6th of September, 1806, the author of "The Spirit of the League," and of "The History of France," breathed his last, preserving the full power of his intellectual faculties up to the very portals of the tomb.

In the above phrase,—"*a man dying, full of life,*" many may see only an ingenious amalgamation of words. But to us it should seem the accurate and forcible expression of a truth, which it would be in vain to contest. It is indeed an evidence of the duplicity of the dynamism in one and the same individual; a proof of the union of two active causes simultaneously created, hitherto inseparable, and the survivor of which, is the biographer of the other.

The imaginary return of the aged to the condition of the infant, has so many charms for a certain sect, that its more zealous members have represented it under a variety of aspects. You may possibly have noticed the following passage from Cabanis :—" *In old persons, the feebleness of the brain, and of those functions which originate therein, gives to their determinations the same mobility, the same characteristic uncertainty, which they possessed during childhood. Extremes resemble one another.*"

Now, this alleged resemblance is but another popular delusion. In enouncing it, the author has not hesitated to advance a theory, which of itself thoroughly overturns materialism. The fundamental principle of organicism is, that the vital force is the result of organization. But if the vital force of the aged and that of the infant be identical, their respective brains should be so likewise. It happens, however, that (as anatomy teaches us) the brain of the latter is remarkable for its softness, while, on the other hand, that of the former is of excessive consistence. We must, therefore, allow that vital force so alike, cannot be the *necessary physical* result of organs in themselves so dissimilar.

Not content with propagating this doctrine relative to the second childhood of the aged, by hardy assertions, exaggerations, theories, &c., an attempt has been made to represent the same through the medium of the æsthetic arts. I have already alluded to that satire upon human existence which is to be found in the writings of Shakespeare. The idea there embodied has been reproduced by the art of the painter. The "Magasin Universel" has lately published one of these attempts, and with such success, that I now never meet an artist who is either ignorant of it, or who does not praise it in the highest terms. This obliges me to examine, along with you, both the idea itself, and the manner in which art has expressed it.

And first, let us glance at the description of human life, as given by Shakespeare, and at the manner in which a modern sculptor has represented it, through the instrumentality of his art. A short prologue which precedes the translation in question, will suffice to show the zeal with which a so-called philosophic sect labours to disseminate this second childhood of the aged, and with it, doubtless, the doctrine of complete annihilation.

"THE SEVEN AGES—A BASSO-RELIEVO AFTER SHAKESPEARE.

"Among the most remarkable of the poetic descriptions of Shakespeare may be cited the piquant and philosophic tirade of Jacques, relative to the different periods of man's existence, in the play of 'As You Like It.' This passage has suggested a beautiful specimen of modern sculpture, greatly admired in one of the expositions held at Somerset House, London. We shall first give a translation of the Shakesperian description, and then institute a comparison between the idea of the poet and the representation of the same in sculpture, as emanating from the studio of M. Behnes.

"All the world's a stage,
And all the men and women merely players;
They have their exits, and their entrances;
And one man in his time plays many parts,
His acts being seven ages. At first the infant,
Mewling and puking in the nurse's arms;
And then the whining schoolboy, with satchel
And shining morning face, creeping like snail,
Unwillingly to school. And then the lover,
Sighing like furnace, with a woful ballad
Made to his mistress's eyebrow. Then the soldier,
Full of strange oaths, and bearded like the pard;
Jealous in honour, sudden and quick in quarrel,
Seeking the bubble reputation
Even in the cannon's mouth. And then the justice,
In fair round belly with good capon lined;
With eyes severe, and beard of formal cut;
Full of wise saws and modern instances;

And so he plays his part. The sixth age shifts
Into the lean and slippered pantaloon;
With spectacles on nose, and pouch on side;
His youthful hose well sav'd, a world too wide
For his shrunk shank; and his big manly voice,
Turning again towards childish treble, pipes
And whistles in his sound. Last scene of all,
That ends this strange eventful history,
Is second childishness and mere oblivion,
Sans teeth, sans eyes, sans taste, sans everything."

I know not whether the above description of human existence, when placed in the frame from which it has been withdrawn, be capable of producing a remarkable effect or not. Certain it is, that there is nought but what may acquire some merit from the objects amid which it may happen to be located. But, in my eyes, this isolated extract is but an insipid, frigid caricature, without point, without instruction. Nor indeed am I exactly aware as to what may have been the design of Shakespeare in stringing together such a succession of incoherent details. It may have been intended as a history of the different ages of a particular individual, but it certainly is not a faithful delineation of Man during the successive stages of his terrestrial existence.

With regard to the sculptor himself, it is evident that he has not merely considered the matter in an æsthetic light; he has endeavoured to moralize as well as to philosophize. A glance is sufficient to prove the fact, and one of his admirers endeavours thus to fix our attention on the profound idea which appears to have animated the artist:

"The form of the basso-relievo is circular, persons of all ages being grouped around. M. Behnes (says he) has chosen the circular form, in order to unite the extremes, or, in other words, to place in close proximity the child just born and the aged man now scarcely conscious of his own existence. The two meet at the foot of a tomb—a truly philosophic idea; upon this tomb is inscribed the Latin quotation, '*Mors janua vitæ*,'—Death is the portal of life."

In truth, on one side of the monument may be seen a fœtus enveloped in its membranes, after it come five babes, each somewhat older than the other; beyond is a young man pondering over a half-closed volume, while his wife and child stand behind.

Further on we perceive a warrior braving every danger, in order to plant a standard. Fame is rewarding him with a laurel crown.

To the right, the descending series of figures is composed, first, of a group of three persons, a judge on his bench, an allegorical representation of justice, and a fettered criminal; to this succeeds an aged individual, attentively examining a page on which some figure is represented, and lastly, a decrepit old man, sitting down almost as closely enveloped in drapery, as the fœtus in its membranes. You see that here the second half of existence is made to decrease alike physically and intellectually: it is, therefore, a tribute to Lucretius.

But I would ask, has the artist attained his object? has he succeeded in clearly demonstrating the *repuerescence* of the aged—his intellectual resemblance to the newborn infant? Has he given me to understand how that death really becomes the gate of life? Has art been able to fascinate me so far as to render similar, two things which I well know to be dissimilar? No, far from it; art has just contradicted, that which the artist would wish to convince me of.

Of one thing I am persuaded, in spite of the artist's project, and that is, the dignity of the latter half of human existence, which is invariably intellectual, and not subject to declension, as compared with the first half. The naked figures on the left, all in the ascending scale, merely show motion, brute force, generative power. Those in the descending scale, all decently clad, evince only intellectual, moral, or scientific tendencies. Say what you will, the first half represents the preponderance of the vital force and its aggregate material, with but little evidence of mature wisdom. The second half represents the pre-eminence of the understanding, and induces me to suspect the progressive decline of the vital principle, since the artist veils with drapery, the ravages which this power undergoes, in that body once so vigorous and flourishing.

The artist and his admirers think to cast a slur upon the sixth age of Shakespeare, by representing the man as occupied with matters of trifling importance. The commentator says that he is working out his horoscope. But be the subject what it may, thought is engaged, and a thought possesses in the psychological hierarchy, a higher rank than an action without thought for its origin. The studies of this period of life have always as their object the elicitation of truth. Be that object real or chimerical, be the method employed logical or not, be the *ultimatum* sought for, present or future happiness . . . . . the intention is at least always elevated and noble, nor can I ever believe that such occupation is inferior to that of a young man indulging (it may be) in immoral reveries. As yet, therefore, I see no tendency to annihilation.

But the artist may imagine that I cannot fail being convinced of such a catastrophe, when he points out to me the inaction of utter decrepitude; he may suppose that by approximating the representation of the seventh age to that of the child still enveloped in its membranes, I may be led to believe that two individuals, in spite of their corporeal differences, can yet be identical as regards their intellect. But there is in reality nothing that authorizes me to credit such a resemblance. Imbued with the tenets of Lucretius, the artist presumes that a decrepit and aged man is of necessity fatuous, yet even the great mass of mankind know well enough that such is by no means the case. The sculptor should by rights have previously informed me of the imbecile condition of the man he represents, otherwise what reason have I for supposing that the one in question, no more possesses the power of thought and reflection than does the fœtus? Why should I imagine that he is but a living automaton, when so many in the same condition have been pregnant with original ideas, with logic, reason, and moral sentiment? He is silent, he makes no sign. It may be so, but he may think not the less. What is to prevent me from believing that it is Theophrastus, finishing his "Characters" at the age of 99, or meditating his last adieus to Lastenia, from whom he is still anticipating a visit. (*Voyage d'Antenor*, chapter xi.)

Why may I not believe the figure to represent the illustrious St. Jérôme, full of learning, genius, and faith, preparing himself, at the age of 88, to receive the last supper?

Why may I not believe it to represent Ruysch, meditating, at the age of 93, over the formation of a new anatomical collection, similar to that which he had just disposed of to a foreign potentate? . . . . . Why may I not believe it to represent Morgagni, aged 89 years, pondering over the information he had received relative to the sensation produced in the medical world, by his great work "On the Seat and Causes of Disease," a work which he had published but a few years previous?

Why may I not believe it to represent the Duke of Nivernois, who, at the age of 82, perceiving death rapidly approaching, wrote a poetic epistle to the physician, dissuading him from seeking a consultation upon his case?

Again, why may I not believe it to represent the academician Teissier, who died at the age of 97, and over whose grave M. Merat thus spoke: "Teissier has run a long career, appreciated and beloved by all who knew him. The gentleness of his manners, the suavity and amiability of his disposition, fully justified the attachment of which he was a general object; in him old age presented nought but what was pleasing, while the charms of his conversation and of his past recollections appeared to increase with his years."

Is it sufficient, then, to be aged and decrepit to be forthwith looked upon as fatuous? Not more than being idiotic would suffice to be considered aged.

What value, then, must we attribute to this philosophic sculptural representation, which strives to place upon an identical footing the new-born infant and the hoary head of age? Will common sense permit of our assimilating the two? . . . . . Have the moral sentiments produced within us by such beings, aught of resemblance?

"*Mors janua vitæ;*" such is the inscription graven upon the tomb, at the foot of which the first and seventh ages are represented as meeting . . . . I could pardon the quotation, if man were in a condition identical with that of the silk-worm, and that the day of his death were that of the birth of his descendants. But no, man creates his like only while in the full enjoyment of his vital powers, and shortly before the divergence which takes place in the respective progression of the two principles.

In what scientific acceptation, however, might we say that "*Death is the gate of life?*" I know of but two—the one which tells us that the same chemical elements may serve to form a variety of successive material aggregates; the other, which shows us how the decomposition of a corpse gives rise to a multiplicity of worms. But these truths are so common and so ordinary, as to be unworthy of typification in a philosophic or poetic point of view.

From the preceding facts, and the deductions which may be drawn from them, you will I think be convinced that the intellectual existence of man is endowed nominally with an indefinite insenescence, and that the principle of intelligence may even be witness of the extinction of the vital force, its congenitor; while, should it happen that a species of somnolence, of short duration, prevents the understanding from beholding the last moments of life, there would still be much difficulty in explaining the same, were causes of a physical nature alone sought for in elucidation of the fact.

The integrity of the intellectual principle amid the ruins of its old abode, is that which attacks, most directly, the doctrine of the Materialists. Cabanis endeavours to ward it off by hardy amphibological assertions, thrown out at random, and utterly wanting in doctrinal value. Now, according to Cabanis, "*the aged man thinks but of himself.*" Looking upon egotism in a particular point of view, La Rochefoucault will tell us whether such a tendency be exclusively that of the aged man. But considering it in the ordinary acceptation, you merely calumniate him, inasmuch as he is frequently occupied in thinking of those about him; his children and pupils may serve to bear witness of this. If, for instance, Monthyon were over-careful of himself during the prime of zoonomic life, his will would absolve him of his old failing, affording as it does ample evidence of love for his fellow-creatures. "*The aged think with difficulty.*" It happens, however, that the world at large tacitly allows the thoughts and ideas of the aged to be the most substantial, the most fruitful, and most worthy of remembrance and respect. In the programme of the fête dedicated to the Supreme Being and instituted by the Convention, may be found the following:—"In the midst of the people, appear their representatives; they are surrounded by *infancy* adorned with violets; by *youth* with myrtle; by *virility* with oak; and by *age* with olive and vine leaves." It is unnecessary even to hint at the conventional value of these different emblems. Again, M. Jubinal, wishing to compare the Rhone and the Escaut in relation to the utility of each, compares the former, impetuous, angry, and rapid, overturning everything in its course, doing as much harm as good, to an impetuous, headstrong youth; and the latter, tranquil, slow, and majestic, a source of industry and riches, to a hoary sage.

Is not such testimony sufficient to counterbalance the assertion of Cabanis: "*The aged man requires repose*"? Physical repose, truly; this to him is indispensable, in order to retard the destruction of his corporeal system, the crumbling away of which he is but too sensible of. Doubtless it is he alone who can regard *eternal repose*, viewed in this light, as a sovereign good. But does he alike desire intellectual repose? no, certainly; it does violence to his feelings, it is his mortal enemy. For him no pleasures now remain, save those derived from new combinations of ideas, and from mental communion with his own thoughts, past and present. Can such enjoyment be designated repose?

Do we perceive any desire for repose in the following extracts from M. Clavering?—"Walter Scott conversed with facility, fluency, and vivacity. The traveller, Simond, and the metaphysician, Bonstettin, might well have entered the lists with him on this point. Well do I remember the eccentric Bonstettin at the age of 87; lively, sparkling, sprightly, coquetting with young girls, and invariably confining his attentions to the prettiest. A pardonable weakness, after all, and amply compensated for by his good humour, his wit, and his general information. At 70 years of age he learnt the Danish, Icelandic, and Celtic languages. Did this indicate a desire for repose?

It is well known that the celebrated Athenian lawgiver, Solon, when at the point of death, and hearing his friends conversing together in an under tone, demanded their reason for so doing. Expressing their surprise at such a question, under such circumstances, he replied, "I merely wished to know the subject of your discussion before I die." (*Val. Max.*, lib. viii. 21.) Do not, however, imagine that such a trait pertains but to a single individual, it is the expression of a characteristic, common to all men whose minds are stored with a certain amount of ideas.

"*The aged aspire only to the termination of their existence.*" (*Cabanis.*) *To aspire* would mean that they are impatient for the annihilation of the intellectual principle. But this is false; there is not an aged man, who, provided his mind and body be in health, does not feel a shudder pass through him at the thought of complete annihilation. An unhappy existence, of which almost every hour brings with it a loss, a privation, a pain, a remorse, may perchance engender the wish to die, with a view to the enjoyment of a happier existence, or the return to a condition of inanity. The loftiest intellect even may *aspire* to such a termination. But this aspiration, the result of mature deliberation, and of which the human understanding is capable at every age, has nothing in common with a tendency to extinction, as though the intellectual principle were as indifferent to such a termination as is the vital force.

You must surely all perceive the repugnance of the intellectual principle in the aged to the idea of its own complete destruction, when, witnessing the demolition of its quondam tenement, it shows forth as strong, as brilliant as ever. What are so many systems of rational psychology, so many divers creeds imagined by those to whom the Almighty has not vouchsafed the knowledge of the true one, but continuous glimpses of the future, which have pre-occupied the imagination of the aged, and which prevent them from reverting to an importunate and repugnant idea—that of annihilation?

I have not here undertaken to examine into all that may happen to the human intellectual principle after death, since the domain of our science is limited by that event. But I denounce, as fallacies, the ordinary acceptation relative to the annihilation of the intellect in the aged. The motives of which I have just spoken, and my own observations, are alike opposed to such an assertion. Never have I seen an aged individual aspiring to annihilation, but I have witnessed those imbued with the idea, living in the agonies of despair. A few Materialists have indeed appeared to meet their fate with resignation; but to be resigned is not to be satisfied or desirous of its approach; and so far from this disposition of the mind supposing a tendency to extinction, it, on the contrary, indicates a sublime power and integrity.

As far as my own experience goes, the frame of mind most common to those who have not enjoyed the blessings of faith, has been one of scepticism. These persons, as they became advanced in years, were daily more and more convinced of the contrast existing between the senescence of the vital force and the insenescence of their own intellectual principle; such conviction showing how each succeeding year served to weaken their preconceived notions relative to the annihilation of the mental powers.

The examples which I have lately brought forward of the insenescence of the human intellectual principle at the most advanced periods of life, have been so numerous, that to adduce more would be unnecessary and useless. To this *argumentum ad judicium*, which should in itself suffice, I shall add another of a different kind. The world at large, without exception, shows by its actions that it accepts this doctrine, if not textually, at least tacitly; following in this respect a line of conduct which comes into direct collision with the dogmas of Lucretius and Cabanis; and I trust that my auditors will, after mature reflection, no longer deny that insenescence of which I speak, lest they find themselves opposed to the majority of the human race, and, at the same time, thinking that which their own actions contradict. These two arguments, *ad vere cundiam et ad hominem*, it is necessary to consider somewhat fully.

1st. *A respect for the aged* constitutes a species of law, which has existed from the remotest periods, flourishes in every civilized community, and forms part and parcel of the public morals. It is not indeed a *natural law*, one derived from a sentiment of humanity, or of such a kind as was styled by Cicero, *innate*, not *instituted*. Nevertheless, being almost as unexceptional as natural laws themselves, it must be derived from a feeling of consideration, itself deduced without effort from the nature of things. What therefore can have implanted this sentiment of respect for age in the members of civilized communities, if not an unreflecting conscious persuasion of the increasing value of the intellect in advanced life?

Let it not be objected to this, that the sentiment is not one of respect, but rather of compassion for the weak, or of gratitude for past services. A respectful consideration for an equal, is not to be confounded with the above feelings. The respect of which I speak, is accompanied by a certain deference, by a conviction that he who is the object of it merits the preference, and by a wish to obtain his approbation.

Consult now your own feelings, and you will perceive that your consideration for an aged man is not proportionate to the number of years he may have attained, but to the sum of intellectual acquisitions which he has made from among the most enlightened classes. An old man, whose station in life presupposes a condition of profound ignorance, or premature fatuity, will not elicit a sentiment much higher than that which we possess for a beast of burden, that has long and faithfully served us, and which we lack the courage to put an end to. But the hoary sage, placed in the same intellectual sphere as yourself, whose education has in all probability equalled your own, and who, at your age, may have resembled you . . . . such an one will inspire respect, since you cannot but feel that you are his inferior. You are not tempted to affect an equality with him, and in this, not only are you obeying certain conventional rules, but listening to a prudent and self-speaking interestedness which warns you from attempting to risk a competition. The dogmas of Lucretius and Cabanis may have told you that the sexagenarian is but your equal mentally speaking, since he is as much beyond the culminating point as you are on this side of it, but you believe it not. A knowledge of the world has taught you the reverse; here, as in many other circumstances, *experience outstrips science*, and what experience has taught you is simply this—that the intellectual principle of the aged man has not returned to that level at which you now stand, and that from its peculiar progressive tendency you cannot possibly determine whether your own mental powers will ever be similar to his.

At all times, and in every age, it is and has been deemed advisable, that a body of men, whether assembled for political, religious, or scientific purposes, should have an individual, or body of individuals at their head, as a source of reference. In the earlier ages even, these were composed of men in whom the decay of the vital force had already become evident, as if wise resolutions were only to be expected from the aged. Is not this a tacit confession of my proposition, that the intellect may remain unscathed, while corporeal senescence is even rapidly advancing?

The mere name of Senatus, amongst the Romans, shows that the individual members of this body had already past their meridian. True, Sigonius speaks of senators who were admitted at the age of 30; but it is unnecessary to discuss the question as to whether these elections were illegal, and whether they were the result of an infraction, or simply of toleration; should such exceptions have been the recompence of superior talents and acquirements, or intrusions achieved by bribery and corruption: it is sufficient for us to remember the original spirit of the institution, designated as it is by the name.

It is true that in the Roman Senate there was a decree, that no one should be entitled to become a member after the age of 60. But it was implicitly agreed, that the interval between 50 and 60 was a period of life more calculated to inspire confidence, in discharging the senatorial duties, than the interval between 20 and 30. Besides which, the period of disqualification was not determined upon from any preconceived notion relative to the decay of the intellectual powers: it was instituted rather in reference to the aspiration of a numerous youth, burning to enjoy the honours and advantages attached to public office, and whom it was not expedient to retain too long in suspense and abeyance. Moreover, the superiority of the mental power in advanced life was not only recognised but taught; and Cicero himself says—"*that in the administration of a republic we should act as in the management of a ship, in which the younger and more active are employed as sailors, while the aged, men of deliberation and resolution, are preferred as pilots.*"

IV. The technicalities of jurisprudence define senescence as "*a weakening of the bodily and mental powers, the result of accumulating years.*" We must presume, therefore, that, legally speaking, the protraction and debility of the vital force do not constitute senescence, unless there be at the same time a weakening of the intellect. As this latter condition, however, is not infallible, it follows that, juridically speaking, senescence is but an accident, an eventuality, and this again agrees with my proposition.

V. How is it that certain ecclesiastical dignities in the Romish church, requiring the greatest amount of mental capacity, intelligence, and wisdom, such, for instance, as the Episcopate, are not attained until after the culminating point of the vital force? It is evident that we have here again an expression of the fact, that the understanding perfects itself at the time the vital force is beginning to decline.

VI. Another proof of the fact, that the world in general tacitly acknowledges the insenescence of the intellectual principle, is this—that while legislators have considered it indispensable to appoint guardians during a considerable portion of the first half of life, they have never thought of instituting an obligatory guardianship of the same nature for the latter period of existence; . . . . and no one appears ever to have considered this as an omission.

In truth, the law has certainly not forgotten that portion of human existence of which senescence is an inevitable accompaniment. On this head there exists an explicit dispensation. At 70 years of age, the individual is exempt from corporeal offices, from military service, from the functions of juror, and from the office of guardian. It is evident, therefore, that the legislature has not been forgetful of those who are stricken in years; it has come, as it were, to the rescue and succour of vital senescence. This senescence it alone recognises as resembling the condition of the infant, and as such has exacted of it nought that decrepitude, defect, or infirmity could render incompatible with duties requiring energy and manly vigour.

But the legislature has, on the other hand, carefully eschewed all ideas which might presuppose a belief in the senescence of the intellectual principle. It has exempted no age from the observance of civil and natural laws. Be he a very Methusaleh, the individual is still subject to them. He has no legal guardian, curator, or tutor, he possesses a full share of civic liberty, and is in consequence responsible for all his acts, and punishable for any infraction of law, without being able to plead want of discernment as an excuse for such infraction.

Is not the reason of all this sufficiently evident? Is it not a tacit avowal of the insenescence of the intellectual principle during the different stages of zoonomic senescence even up to the renewal of infantile incapacity. During infancy, such is the helpless condition of the vital force, that a certain amount of protection is necessary until the understanding has become developed and capable of exercising its own rights and privileges. Were the mother's solicitude to be withheld, the infant must perish. Law steps in to the mother's assistance, and the infant is preserved. When age, however, has again rendered the vital principle as powerless as it was during infancy, must the law again intervene, and render the same assistance as formerly? No. The moral principle of the man is in full vigour, and should be the most zealous, the most interested guardian of its own system. It may still point out what is necessary for its conservation, and seek, demand, or implore the same, according to the position which its possessor may occupy in the social scale. If by chance, however, the intellectual principle should become dimmed or oppressed by the supervention of a disease which incapacitated it from thinking or acting aright, then a guardian would become indispensable, and the legislature would not fail at once to appoint a competent one. But as such an event is only casual, the remedy can merely be extemporary. Would *you*, I ask, submit to the dictates of an official guardian, while enjoying the full powers of the intellect, simply because a certain number of years had passed over your head?

To think and act thus, however, we must feel convinced that nought can authorize the belief in a dogma so erroneous, as that the intellectual principle should of *necessity* deteriorate at any given and fixed period of existence, however protracted; or that there can be any specific epoch, at which a citizen, a magistrate, a priest, a bishop, a king, or a pope, should become naturally, and without exception, ineligible for the exercise of those intellectual functions which the title renders imperative.

In conclusion, let us at once admit the *natural and habitual insenescence* of the intellectual principle, in spite of the *senescence* of the vital force, under the penalty of falling into the most absurd contradictions; while we at the same time endeavour to establish an agreement between the science we are striving to illustrate, and that common sense which directs us almost by instinct.

It is now time for us to acquire a clear and comparative view of the intellectual existence of man in relation to his zoonomic life.

The lines by which this zoonomic life may be represented, constitute a figure termed a diangle. We have already observed that intellectual does not commence simultaneously with vital action, and have also shown that there is an interval between the moment of creation and that of primary action; which interval may be represented by a line drawn from the creative point, to that from which the curve commences; thus—

B Action commences.

A The generative point.

We also see that the angle of the diangle, which is that of an ogee arch, thus, does not efficiently and accurately represent the first attempts at intellectual manifestation, and that consequently the origin of mental activity must be represented by a curve, the commencement of which it is difficult to define distinctly; thus—

The curve which represents the progress of the intellectual principle cannot be subject to interruptions or breaks, since the intellect is not liable to variations of increase and decrease, as is the vital force. The two branches of the ellipse, therefore, must be continued during their entire course, or at least interrupted merely by an eclipse, so to speak, if we deem it necessary to represent at the same time those passing alienations to which mental existence is prone.

Having, then, arrived at the meridian of life, what must we do with the curve? Are the lines again to approximate, as in the spindle-shaped figure which we have shown to represent zoonomic life?—No; inasmuch as all I have hitherto said has been to show that the collective value of intellectual existence, far from diminishing or becoming restricted within limits gradually narrowing, increases in vigour and develops itself more and more during a considerable period of vital senescence. The curve, therefore, by which it is represented, must have some resemblance to a parabola, whose extremities continue to separate slowly, in proportion as they become distant from the point of origin.

Here, however, an objection presents itself, which I have no wish to avoid. The branches of a parabola, while separating, naturally increase the amount of space included between them. But we cannot admit that the amplitude of the intellect invariably increases during old age. Allowing that we may not discern an evident diminution in the powers of the understanding, it is at least certain, that in very many instances its psychological value does not increase, and consequently the parabolic form will not give a figurative representation of the truth.

I admit the fact, without, however, renouncing my original idea. I admit that, taken collectively, the faculties of the understanding do not increase—nay, that they even diminish somewhat during the final period of zoonomic life. But it must be borne in mind, that in the exercise of many of these faculties the vital force co-operates; and as, in regard to this latter principle, its intensity of action invariably diminishes during old age, we must allow that an increase, and even a stationary condition, of the understanding, indicates a necessary augmentation on the part of the intellectual principle, since it is forced to execute, unassisted, those functions in which it formerly had a coadjutor. From the moment at which the vital principle begins to slacken in its exertion, does the intellectual principle require to make up for the deficiencies of its colleague. A period then arrives, at which debility renders the vital force powerless, and throws the whole burden upon the intellectual principle. It is easy to conceive that there may be cases in which this excess of activity would be inadequate to achieve what could have been done by the two powers when in conjunction, and yet the survivor may have done more than when actually co-operating with its defunct partner.

I see, therefore, no reason for altering the form of the curve. The parabola will still be the figure best capable of representing the truths which I desire to propagate.

The intellectual existence of man may therefore be imitated by a solid body, resulting from the revolution of an elongated parabola around a central axis, thus—

The generative point A represents the moment of conception. The dotted line extending from this point to the commencement of the curve E represents the latent period of the intellectual principle during intra uterine existence. The curve not uniting again, it is impossible to assign the limits of the parabola; in proportion, however, as its length increases, does the eversion of its including lines become less evident.

Having hitherto represented the two periods of human existence separately, through the medium of linear configuration, let me endeavour to show them in a similar manner, but in a state of combination.

For this purpose I unite the diangle which represents the vital force, and the parabola which represents the human intellectual principle; causing them to interlace in such a manner as at once to demonstrate their mutual relationship in the system which they constitute; thus—

The diangle A B C D indicates the entire duration of life, A and C being its commencement and its termination. The intersecting line B D divides it into two parts. At the commencement of the part marked A is the generative point of a parabola; a dotted straight line starting from it, represents the latent phase of the intellectual principle. The beginning of the curve at E is an emblem of the earliest operations of the intelligence: its branches remain within the limits of the diangle, to remind us that the vital force preponderates over the intellectual principle during the first half of life. At the point of intersection the parabolic lines begin to make their exit from the area of the diangle, and continue to diverge slowly up to the level of the terminal point C of the diangle, at which point life becomes extinct.

A figure of this kind, intended not to represent corporeal objects, but to fix the attention upon abstract ideas, does not require the accurate tracing of a ruler and compass; and I should wish it to become as familiar to you all, as an ordinary letter of the alphabet. A combination of the small Greek *theta*, $\theta$, and *upsilon*, $\upsilon$, with an *iota*, $\iota$, beneath, appear to me sufficient to embody the idea in toto; thus—

A symbolic figure like this may then serve to express the fundamental principles of the science of Man, conceived in an Hippocratic spirit, in opposition to the doctrines of the Materialists, the Cartesians, and the Stahlians. In laying before you these views upon the subject, I have endeavoured to remain strictly within my own sphere, feeling too well my deficiencies, to attempt to trangress its limits, and have therefore reasoned upon facts and inductive philosophy alone, eschewing all *à priori* theory.

I have laid down the *insenescence* of the intellectual principle, inasmuch as it is an ascertained *fact;* but I cannot prove its *insenescibility,* since this is not to be demonstrated by observation or logic—the only means that are at my command.

It is something, however, to have proved that, in the course of human existence, there is but one portion of which we know by experience the beginning and the end; and that the remaining one is unfinished, since we merely see its initial point, and are not even aware whether it be susceptible of a terminal one.

Buffon has said, that the saddest reflection, or, in other words, that which most mars the happiness of man, is the certain prospect of his approaching end, and that this idea is a canker in the heart of every aged individual. At the age of 70 he set about calculating the probabilities of life during the successive years of advanced age, and he thinks to have rendered the aged a real service, by showing them, that at any period a man who is in health may entertain a legitimate hope of yet living several years. "The best use," says he, "that a man can make of his mental powers is to collect around him the images of all that is pleasing, and to ward off all disagreeable objects—more especially those *ideas* which may give him uneasiness; while, to effect this, it is sometimes sufficient merely to regard things in their own proper light."

The service, then, which Buffon has rendered, consists in regarding the proximity of death as always doubtful, whatever be the age of the individual; but there are some to whom such a doubt is of but little consequence, when once aware that their end is inevitable and not far distant. They look merely to that vital principle which is about to escape them, and which has afflicted them provisionally with all the miseries of caducity. But feeling themselves morally the same as heretofore, and dreading annihilation, would they not find hope and consolation in a conviction of that insenescence of the intellectual principle, which daily renders more probable the continuation and imperishability of this principle? When of two self-associated causes, the one has run through its phases of duration and is on the point of achieving its destiny, is it not allowable to remain in doubt relative to the extent of the survivor's vocation, when its possessor is unable to perceive the slightest refraction of its rays—the least convergence in the onward progress of the glorious cone?

Plato, Aristotle, Descartes, Silhon, Leibnitz, and many other philosophers, have endeavoured to demonstrate the immortality of the principle in question. Their premises, however, are all of an *à priori* nature. Such isolated theorems, devoid of experimental basis, cannot be alleged as pertaining to our school, whose philosophy is essentially that of Bacon. On the other hand, I do not hesitate to affirm that the *insenescence* of the intellectual principle, if fully admitted, must tend strongly to favour any theory which should have for its basis not merely the *insenescibility*, but likewise the *immortality* of this principle.

The above idea being susceptible of diverse applications, be not surprised at my associating it with a symbolical figure, equally capable of being multiplied at pleasure, and of which I may avail myself as it were, of an amulet. Not only does it impress on the mind a fact, the deductions from which *are necessary to science, and to our own individual happiness ;* but, further, it will constitute a link in the chain of memory, serving to connect me with those for whom these lessons have been prepared, and who have hitherto been so willing to follow me during their somewhat complex details.

---

## Figures and Tables

**Figure f1:**
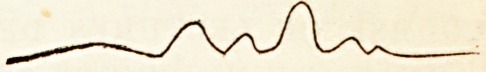


**Figure f2:**
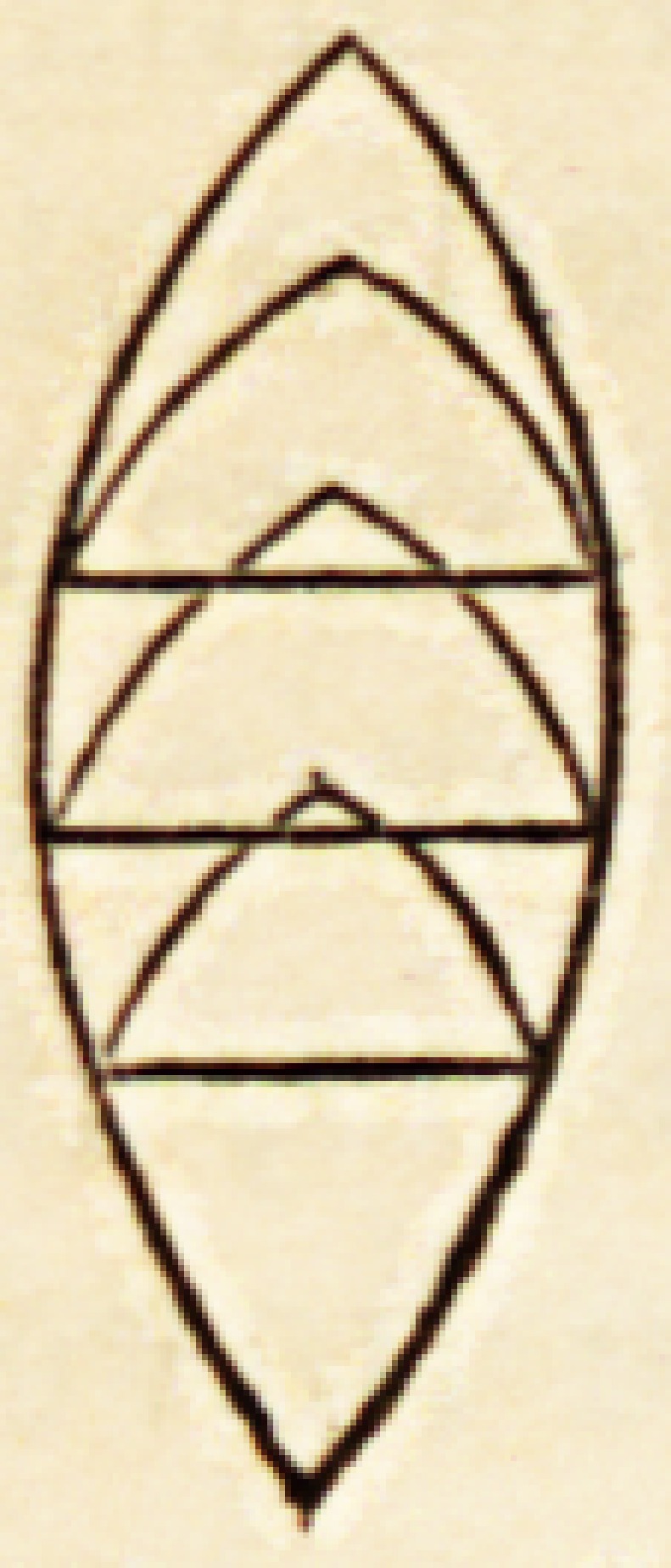


**Figure f3:**
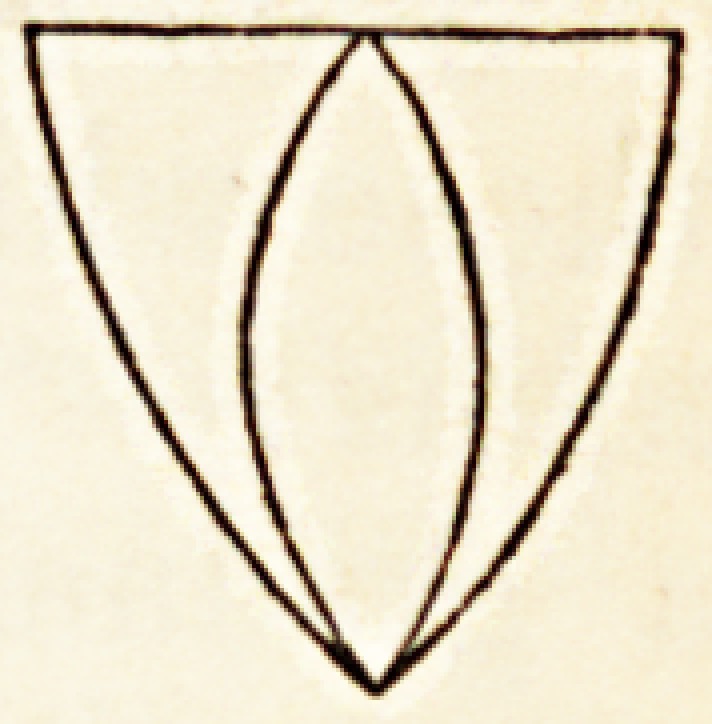


**Figure f4:**
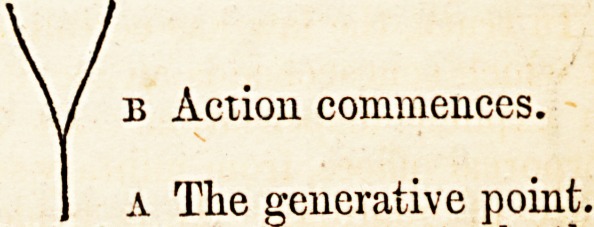


**Figure f5:**
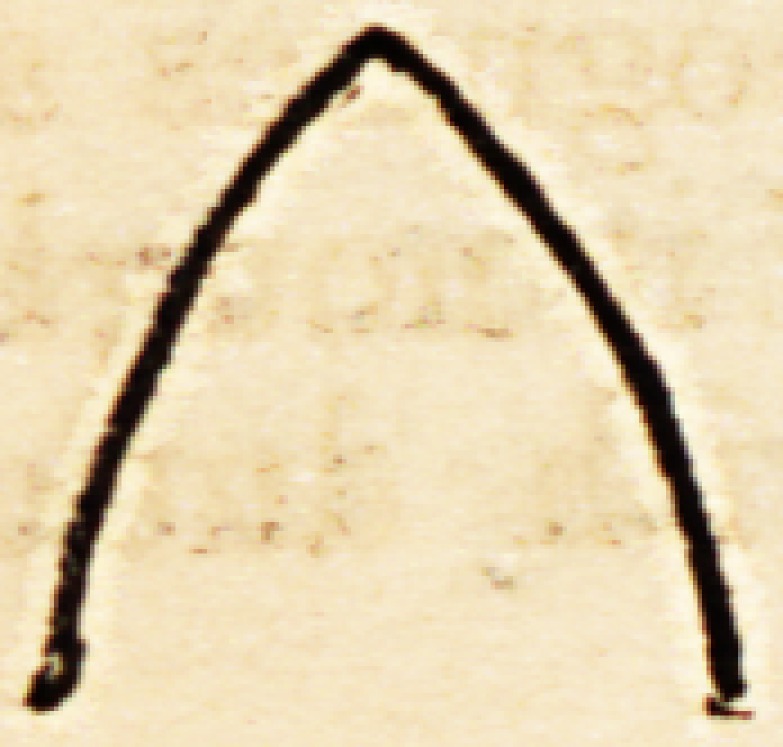


**Figure f6:**
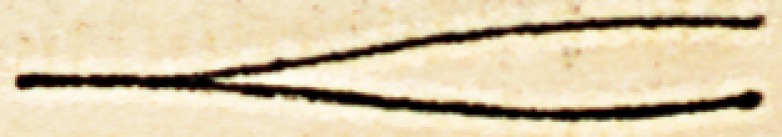


**Figure f7:**
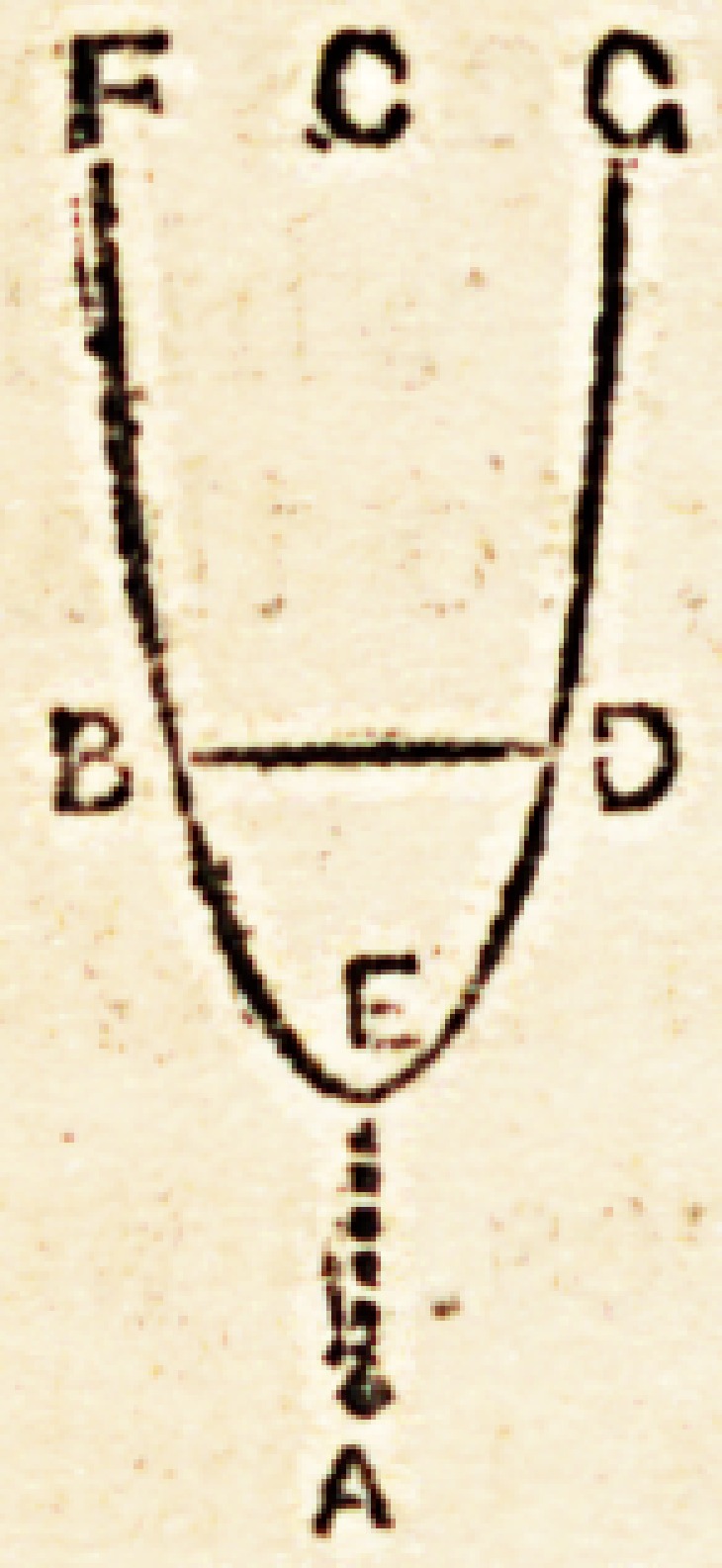


**Figure f8:**
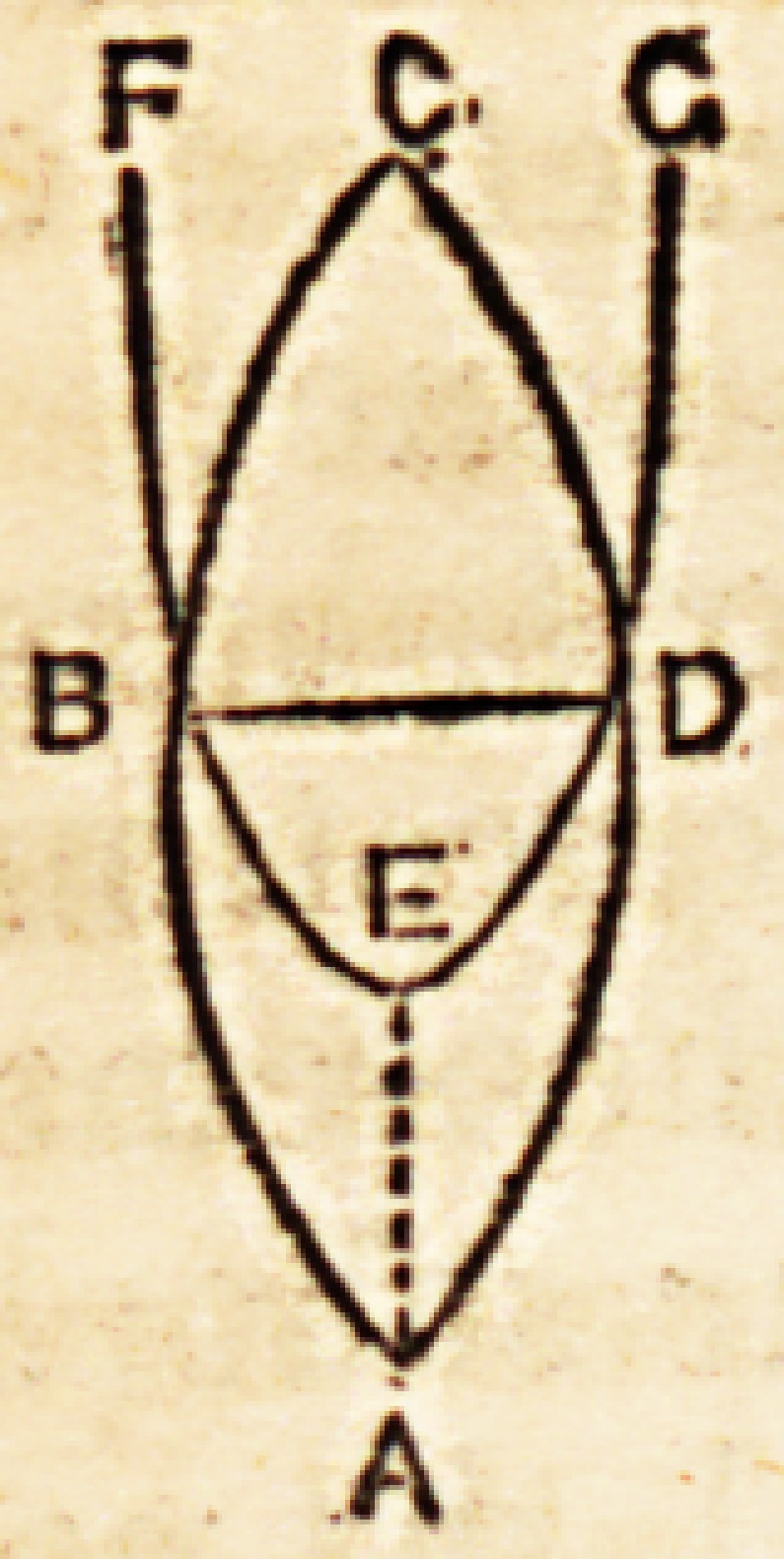


**Figure f9:**